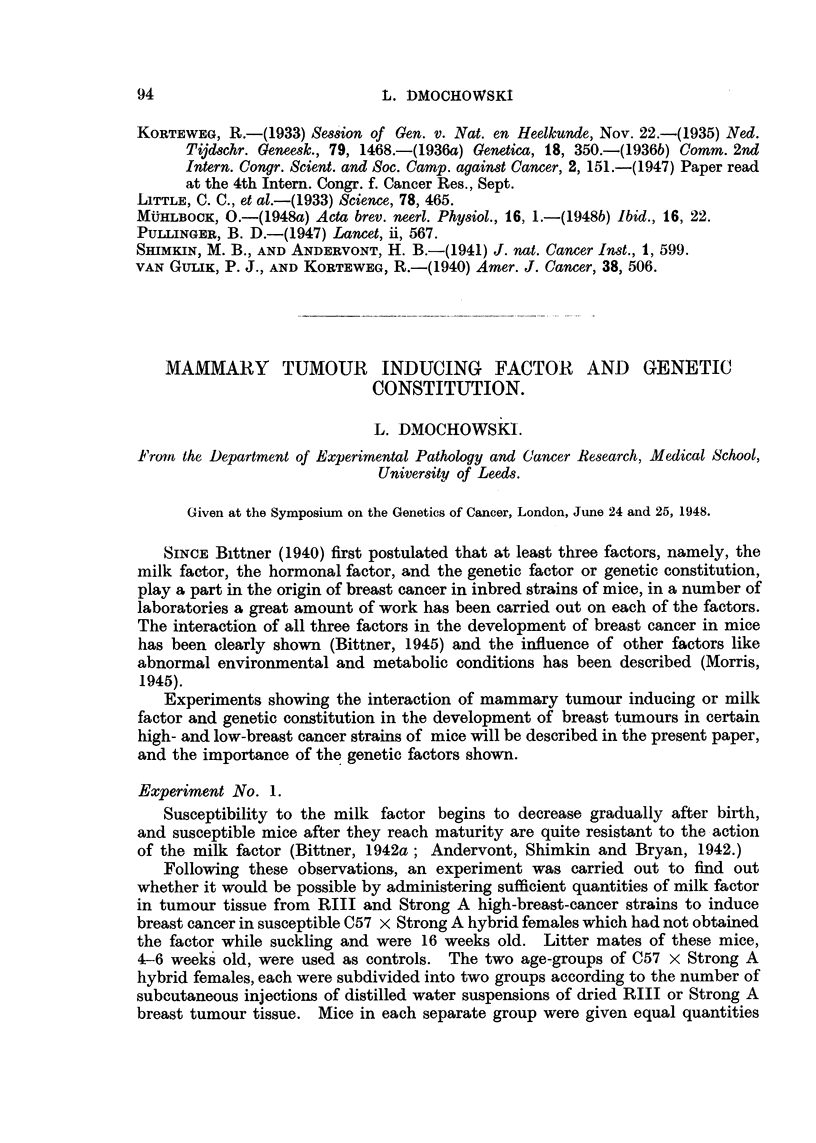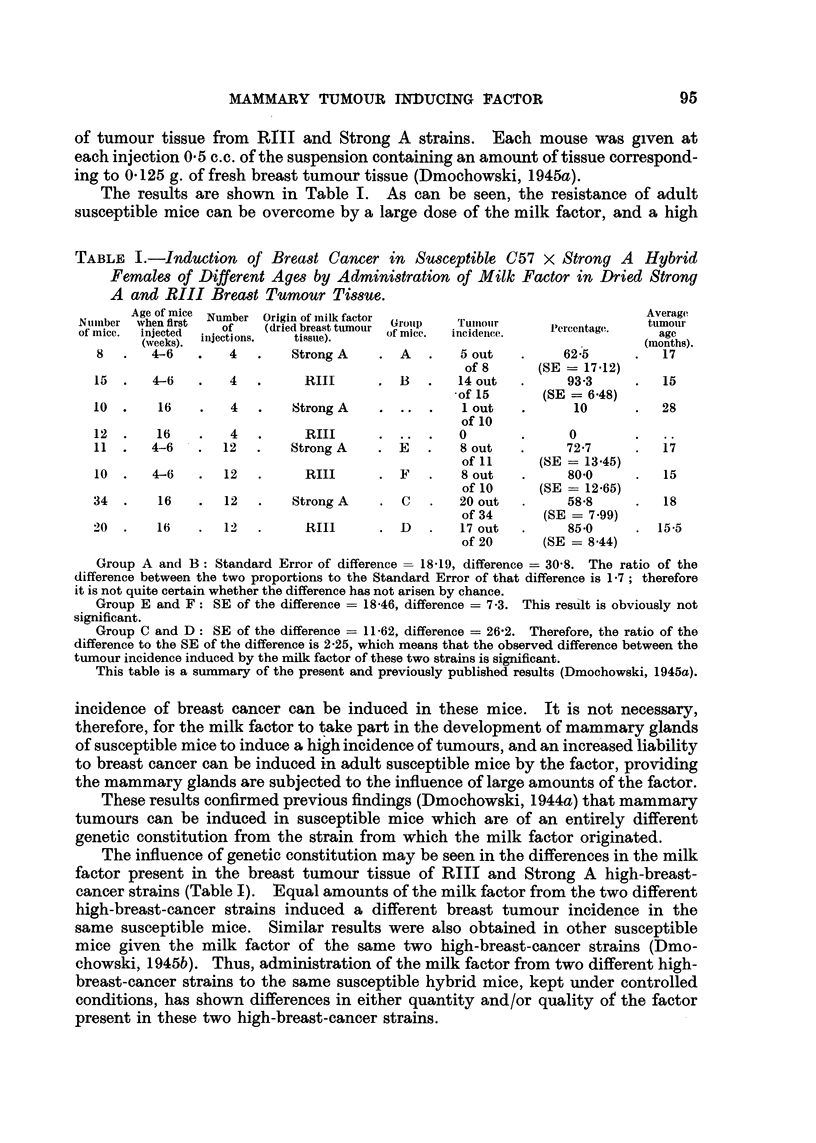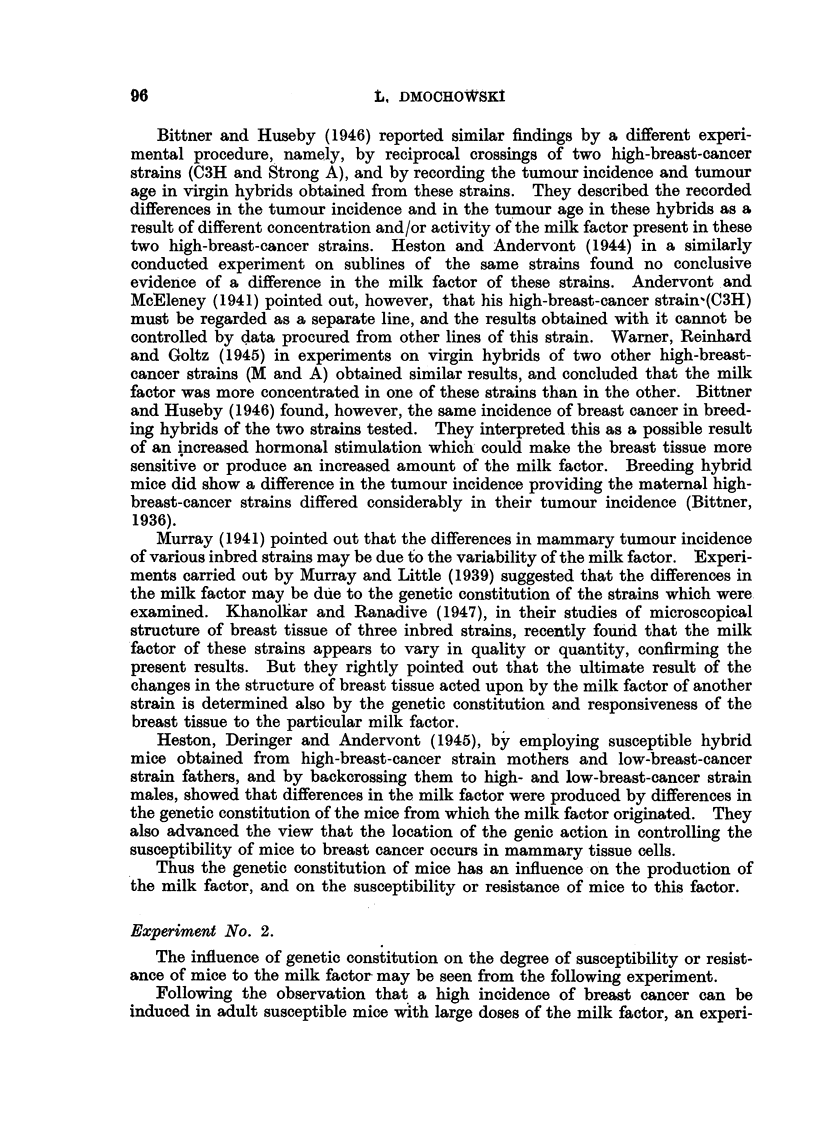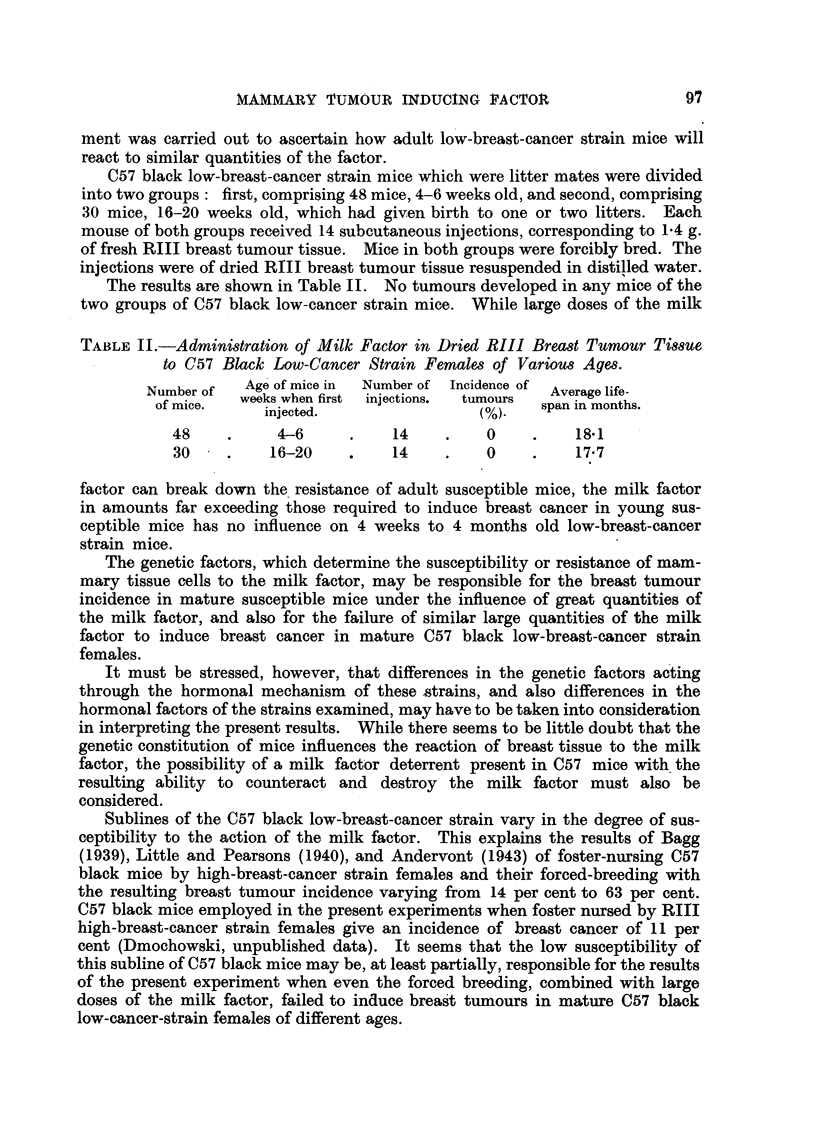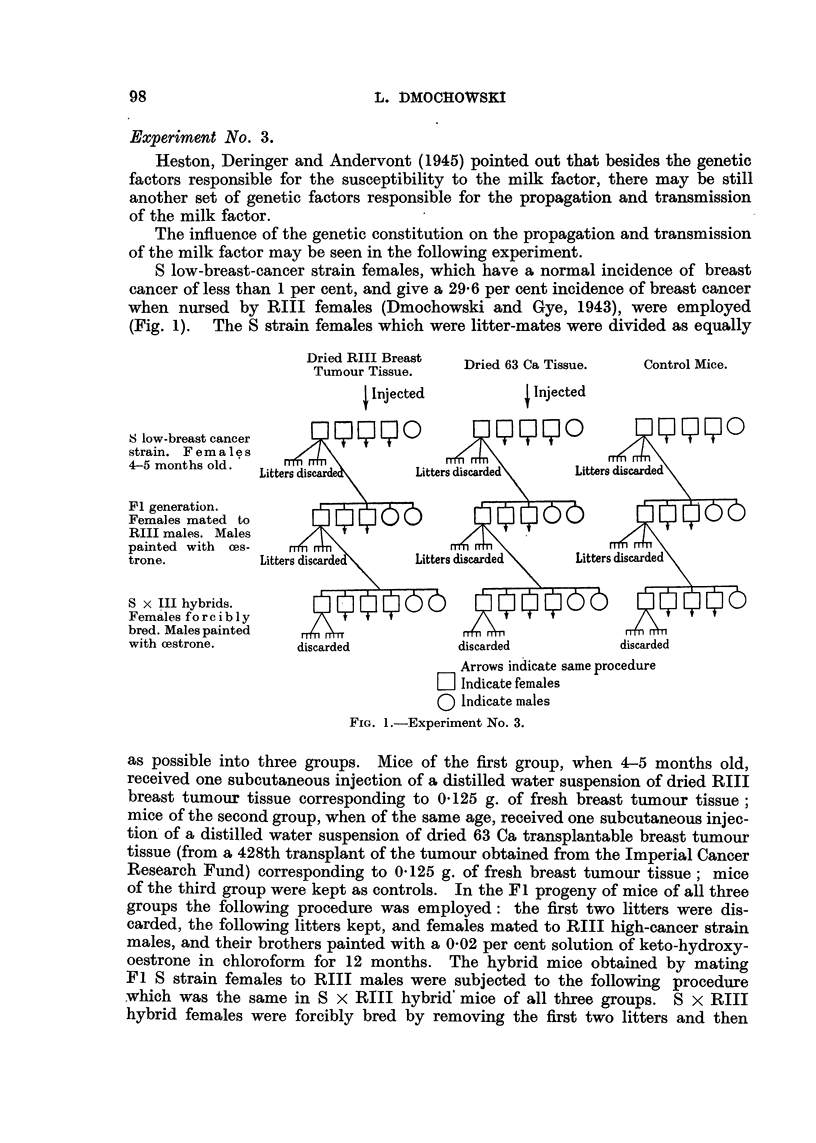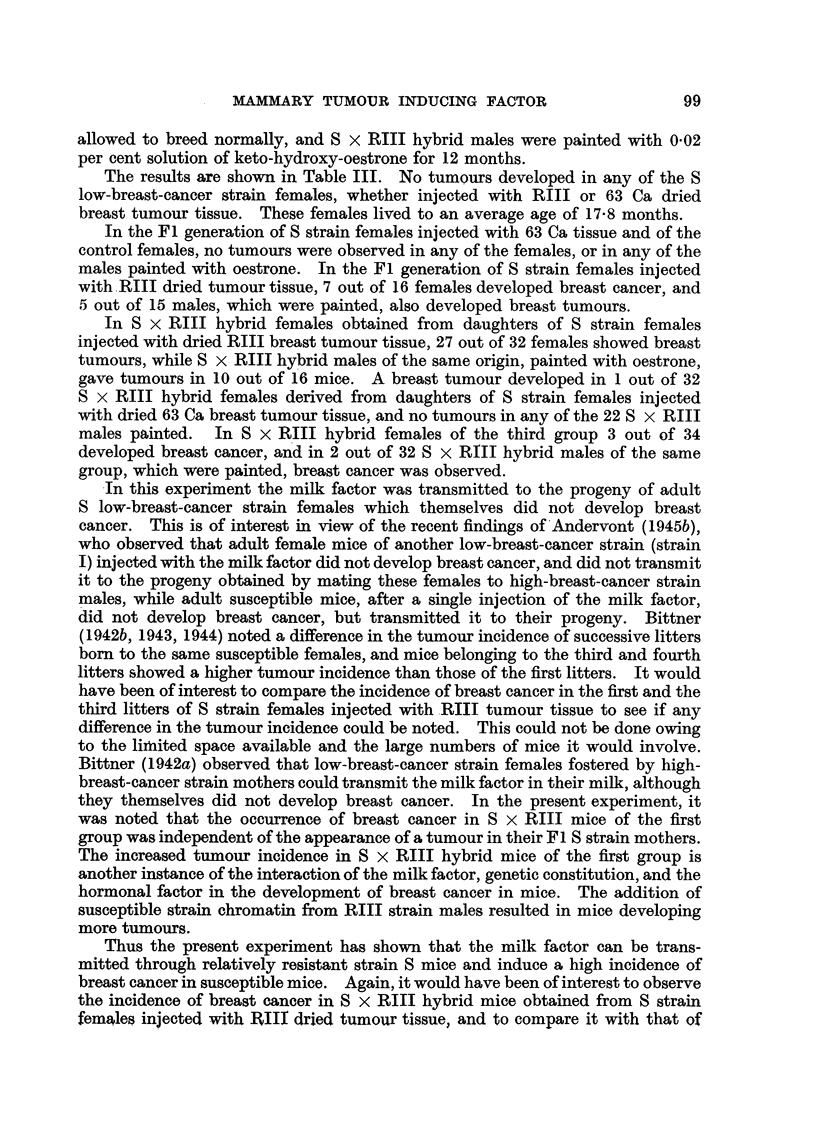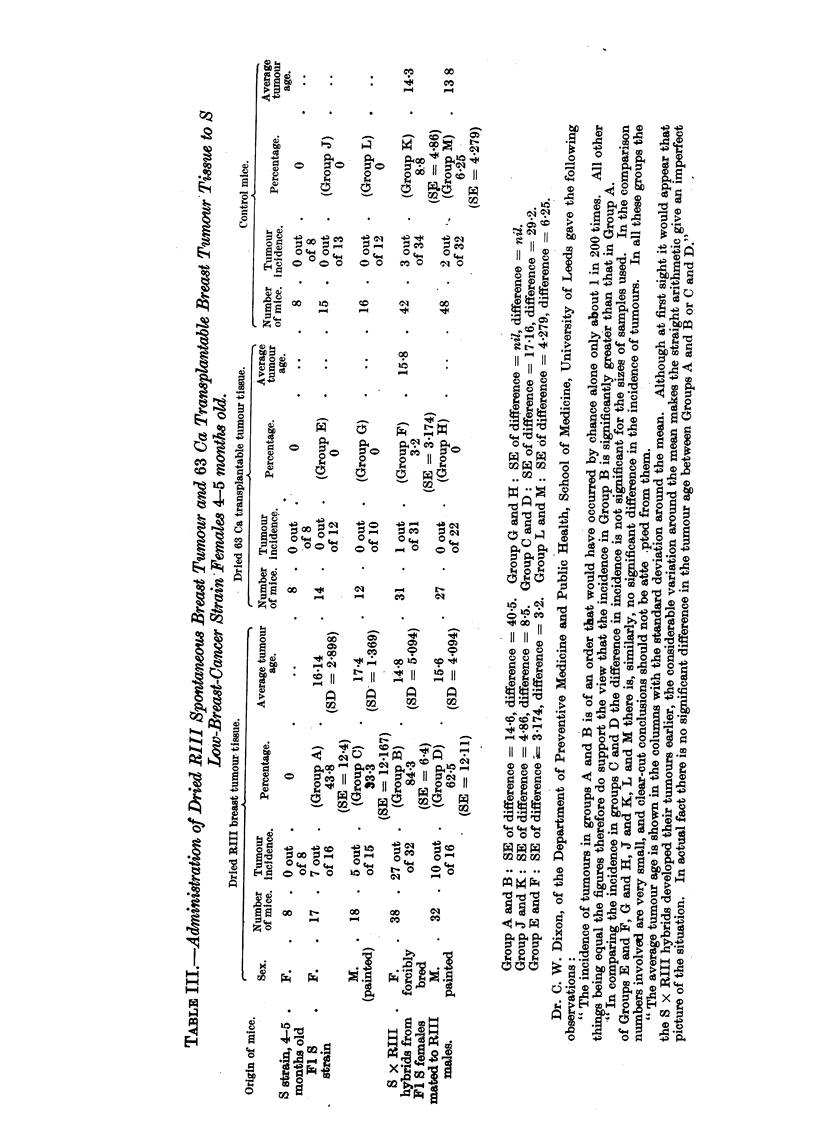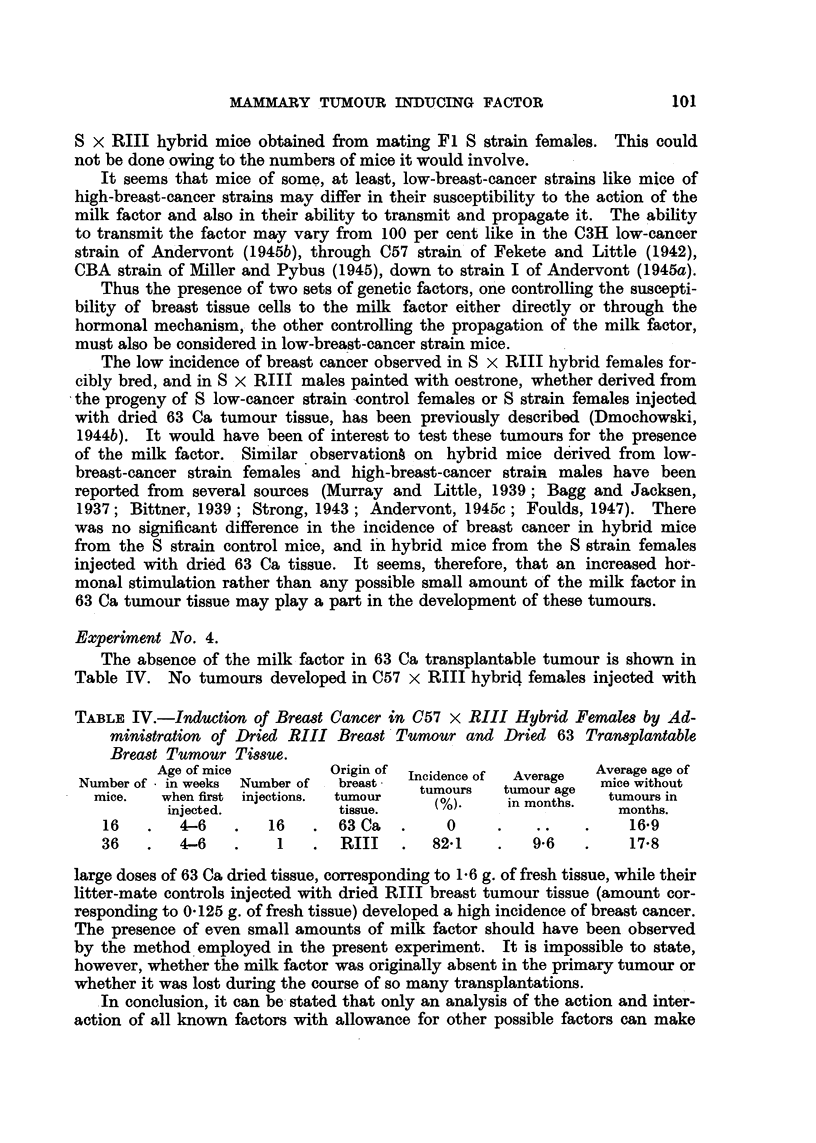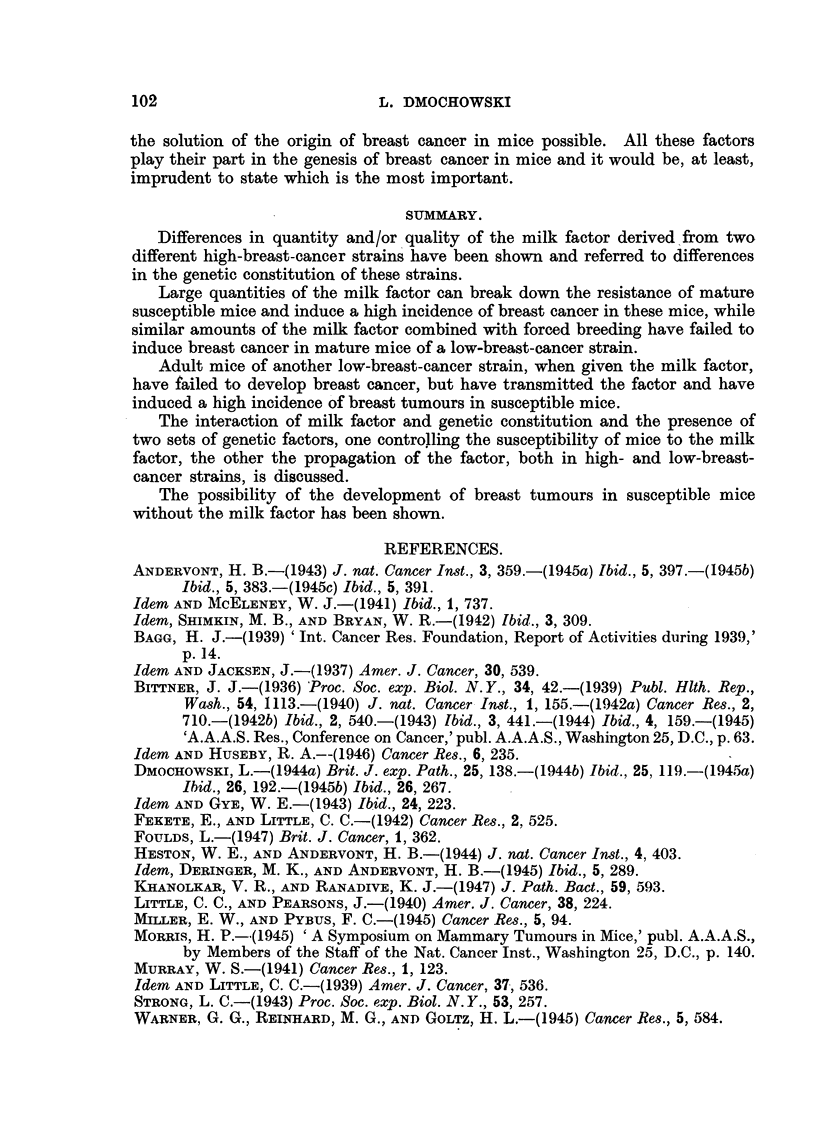# Mammary Tumour Inducing Factor and Genetic Constitution

**DOI:** 10.1038/bjc.1948.13

**Published:** 1948-06

**Authors:** L. Dmochowski


### MAMMARY TUMOUR INDUCING FACTOR AND GENETIC CONSTITUTION.

L. DMOCHOWSKI.

*From the Department of Experimental Pathology and Cancer Research, Medical School, University of Leeds.*

Given at the Symposium on the Genetics of Cancer, London, June 24 and 25, 1948.

SINCE Bittner (1940) first postulated that at least three factors, namely, the milk factor, the hormonal factor, and the genetic factor or genetic constitution, play a part in the origin of breast cancer in inbred strains of mice, in a number of laboratories a great amount of work has been carried out on each of the factors. The interaction of all three factors in the development of breast cancer in mice has been clearly shown (Bittner, 1945) and the influence of other factors like abnormal environmental and metabolic conditions has been described (Morris, 1945).

Experiments showing the interaction of mammary tumour inducing or milk factor and genetic constitution in the development of breast tumours in certain high- and low-breast cancer strains of mice will be described in the present paper, and the importance of the genetic factors shown.

*Experiment No.* 1.

Susceptibility to the milk factor begins to decrease gradually after birth, and susceptible mice after they reach maturity are quite resistant to the action of the milk factor (Bittner, 1942*a* ; Andervont, Shimkin and Bryan, 1942.)

Following these observations, an experiment was carried out to find out whether it would be possible by administering sufficient quantities of milk factor in tumour tissue from RIII and Strong A high-breast-cancer strains to induce breast cancer in susceptible C57 × Strong A hybrid females which had not obtained the factor while suckling and were 16 weeks old. Litter mates of these mice, 4–6 weeks old, were used as controls. The two age-groups of C57 × Strong A hybrid females, each were subdivided into two groups according to the number of subcutaneous injections of distilled water suspensions of dried RIII or Strong A breast tumour tissue. Mice in each separate group were given equal quantities of tumour tissue from RIII and Strong A strains. Each mouse was given at each injection 0·5 c.c. of the suspension containing an amount of tissue corresponding to 0·125 g. of fresh breast tumour tissue (Dmochowski, 1945*a*).

The results are shown in Table I. As can be seen, the resistance of adult susceptible mice can be overcome by a large dose of the milk factor, and a high

TABLE I.—*Induction of Breast Cancer in Susceptible C57 × Strong A Hybrid Females of Different Ages by Administration of Milk Factor in Dried Strong A and RIII Breast Tumour Tissue.*

| Number of mice. | Age of mice when first injected (weeks). | Number of injections. | Origin of milk factor (dried breast tumour tissue). | Group of mice. | Tumour incidence. | Percentage. | Average tumour age (months). |
|---|---|---|---|---|---|---|---|
| 8 | 4–6 | 4 | Strong A | A | 5 out of 8 | 62·5 (SE = 17·12) | 17 |
| 15 | 4–6 | 4 | RIII | B | 14 out of 15 | 93·3 (SE = 6·48) | 15 |
| 10 | 16 | 4 | Strong A | .. | 1 out of 10 | 10 | 28 |
| 12 | 16 | 4 | RIII | .. | 0 | 0 | .. |
| 11 | 4–6 | 12 | Strong A | E | 8 out of 11 | 72·7 (SE = 13·45) | 17 |
| 10 | 4–6 | 12 | RIII | F | 8 out of 10 | 80·0 (SE = 12·65) | 15 |
| 34 | 16 | 12 | Strong A | C | 20 out of 34 | 58·8 (SE = 7·99) | 18 |
| 20 | 16 | 12 | RIII | D | 17 out of 20 | 85·0 (SE = 8·44) | 15·5 |

Group A and B : Standard Error of difference = 18·19, difference = 30·8. The ratio of the difference between the two proportions to the Standard Error of that difference is 1·7 ; therefore it is not quite certain whether the difference has not arisen by chance.

Group E and F : SE of the difference = 18·46, difference = 7·3. This result is obviously not significant.

Group C and D : SE of the difference = 11·62, difference = 26·2. Therefore, the ratio of the difference to the SE of the difference is 2·25, which means that the observed difference between the tumour incidence induced by the milk factor of these two strains is significant.

This table is a summary of the present and previously published results (Dmochowski, 1945*a*).

incidence of breast cancer can be induced in these mice. It is not necessary, therefore, for the milk factor to take part in the development of mammary glands of susceptible mice to induce a high incidence of tumours, and an increased liability to breast cancer can be induced in adult susceptible mice by the factor, providing the mammary glands are subjected to the influence of large amounts of the factor.

These results confirmed previous findings (Dmochowski, 1944*a*) that mammary tumours can be induced in susceptible mice which are of an entirely different genetic constitution from the strain from which the milk factor originated.

The influence of genetic constitution may be seen in the differences in the milk factor present in the breast tumour tissue of RIII and Strong A high-breast-cancer strains (Table I). Equal amounts of the milk factor from the two different high-breast-cancer strains induced a different breast tumour incidence in the same susceptible mice. Similar results were also obtained in other susceptible mice given the milk factor of the same two high-breast-cancer strains (Dmochowski, 1945*b*). Thus, administration of the milk factor from two different high-breast-cancer strains to the same susceptible hybrid mice, kept under controlled conditions, has shown differences in either quantity and/or quality of the factor present in these two high-breast-cancer strains.

Bittner and Huseby (1946) reported similar findings by a different experimental procedure, namely, by reciprocal crossings of two high-breast-cancer strains (C3H and Strong A), and by recording the tumour incidence and tumour age in virgin hybrids obtained from these strains. They described the recorded differences in the tumour incidence and in the tumour age in these hybrids as a result of different concentration and/or activity of the milk factor present in these two high-breast-cancer strains. Heston and Andervont (1944) in a similarly conducted experiment on sublines of the same strains found no conclusive evidence of a difference in the milk factor of these strains. Andervont and McEleney (1941) pointed out, however, that his high-breast-cancer strain (C3H) must be regarded as a separate line, and the results obtained with it cannot be controlled by data procured from other lines of this strain. Warner, Reinhard and Goltz (1945) in experiments on virgin hybrids of two other high-breast-cancer strains (M and A) obtained similar results, and concluded that the milk factor was more concentrated in one of these strains than in the other. Bittner and Huseby (1946) found, however, the same incidence of breast cancer in breeding hybrids of the two strains tested. They interpreted this as a possible result of an increased hormonal stimulation which could make the breast tissue more sensitive or produce an increased amount of the milk factor. Breeding hybrid mice did show a difference in the tumour incidence providing the maternal high-breast-cancer strains differed considerably in their tumour incidence (Bittner, 1936).

Murray (1941) pointed out that the differences in mammary tumour incidence of various inbred strains may be due to the variability of the milk factor. Experiments carried out by Murray and Little (1939) suggested that the differences in the milk factor may be due to the genetic constitution of the strains which were examined. Khanolkar and Ranadive (1947), in their studies of microscopical structure of breast tissue of three inbred strains, recently found that the milk factor of these strains appears to vary in quality or quantity, confirming the present results. But they rightly pointed out that the ultimate result of the changes in the structure of breast tissue acted upon by the milk factor of another strain is determined also by the genetic constitution and responsiveness of the breast tissue to the particular milk factor.

Heston, Deringer and Andervont (1945), by employing susceptible hybrid mice obtained from high-breast-cancer strain mothers and low-breast-cancer strain fathers, and by backcrossing them to high- and low-breast-cancer strain males, showed that differences in the milk factor were produced by differences in the genetic constitution of the mice from which the milk factor originated. They also advanced the view that the location of the genic action in controlling the susceptibility of mice to breast cancer occurs in mammary tissue cells.

Thus the genetic constitution of mice has an influence on the production of the milk factor, and on the susceptibility or resistance of mice to this factor.

*Experiment No. 2.*

The influence of genetic constitution on the degree of susceptibility or resistance of mice to the milk factor may be seen from the following experiment.

Following the observation that a high incidence of breast cancer can be induced in adult susceptible mice with large doses of the milk factor, an experiment was carried out to ascertain how adult low-breast-cancer strain mice will react to similar quantities of the factor.

C57 black low-breast-cancer strain mice which were litter mates were divided into two groups : first, comprising 48 mice, 4–6 weeks old, and second, comprising 30 mice, 16–20 weeks old, which had given birth to one or two litters. Each mouse of both groups received 14 subcutaneous injections, corresponding to 1·4 g. of fresh RIII breast tumour tissue. Mice in both groups were forcibly bred. The injections were of dried RIII breast tumour tissue resuspended in distilled water.

The results are shown in Table II. No tumours developed in any mice of the two groups of C57 black low-cancer strain mice. While large doses of the milk

TABLE II.—*Administration of Milk Factor in Dried RIII Breast Tumour Tissue to C57 Black Low-Cancer Strain Females of Various Ages.*

| Number of of mice. | Age of mice in weeks when first injected. | Number of injections. | Incidence of tumours (%). | Average life-span in months. |
|---|---|---|---|---|
| 48 | 4–6 | 14 | 0 | 18·1 |
| 30 | 16–20 | 14 | 0 | 17·7 |

factor can break down the resistance of adult susceptible mice, the milk factor in amounts far exceeding those required to induce breast cancer in young susceptible mice has no influence on 4 weeks to 4 months old low-breast-cancer strain mice.

The genetic factors, which determine the susceptibility or resistance of mammary tissue cells to the milk factor, may be responsible for the breast tumour incidence in mature susceptible mice under the influence of great quantities of the milk factor, and also for the failure of similar large quantities of the milk factor to induce breast cancer in mature C57 black low-breast-cancer strain females.

It must be stressed, however, that differences in the genetic factors acting through the hormonal mechanism of these strains, and also differences in the hormonal factors of the strains examined, may have to be taken into consideration in interpreting the present results. While there seems to be little doubt that the genetic constitution of mice influences the reaction of breast tissue to the milk factor, the possibility of a milk factor deterrent present in C57 mice with the resulting ability to counteract and destroy the milk factor must also be considered.

Sublines of the C57 black low-breast-cancer strain vary in the degree of susceptibility to the action of the milk factor. This explains the results of Bagg (1939), Little and Pearsons (1940), and Andervont (1943) of foster-nursing C57 black mice by high-breast-cancer strain females and their forced-breeding with the resulting breast tumour incidence varying from 14 per cent to 63 per cent. C57 black mice employed in the present experiments when foster nursed by RIII high-breast-cancer strain females give an incidence of breast cancer of 11 per cent (Dmochowski, unpublished data). It seems that the low susceptibility of this subline of C57 black mice may be, at least partially, responsible for the results of the present experiment when even the forced breeding, combined with large doses of the milk factor, failed to induce breast tumours in mature C57 black low-cancer-strain females of different ages.

*Experiment No.* 3.

Heston, Deringer and Andervont (1945) pointed out that besides the genetic factors responsible for the susceptibility to the milk factor, there may be still another set of genetic factors responsible for the propagation and transmission of the milk factor.

The influence of the genetic constitution on the propagation and transmission of the milk factor may be seen in the following experiment.

S low-breast-cancer strain females, which have a normal incidence of breast cancer of less than 1 per cent, and give a 29·6 per cent incidence of breast cancer when nursed by RIII females (Dmochowski and Gye, 1943), were employed (Fig. 1). The S strain females which were litter-mates were divided as equally

FIG. 1.—Experiment No. 3.

as possible into three groups. Mice of the first group, when 4–5 months old, received one subcutaneous injection of a distilled water suspension of dried RIII breast tumour tissue corresponding to 0·125 g. of fresh breast tumour tissue; mice of the second group, when of the same age, received one subcutaneous injection of a distilled water suspension of dried 63 Ca transplantable breast tumour tissue (from a 428th transplant of the tumour obtained from the Imperial Cancer Research Fund) corresponding to 0·125 g. of fresh breast tumour tissue; mice of the third group were kept as controls. In the F1 progeny of mice of all three groups the following procedure was employed: the first two litters were discarded, the following litters kept, and females mated to RIII high-cancer strain males, and their brothers painted with a 0·02 per cent solution of keto-hydroxy-oestrone in chloroform for 12 months. The hybrid mice obtained by mating F1 S strain females to RIII males were subjected to the following procedure which was the same in S × RIII hybrid mice of all three groups. S × RIII hybrid females were forcibly bred by removing the first two litters and then allowed to breed normally, and S × RIII hybrid males were painted with 0·02 per cent solution of keto-hydroxy-oestrone for 12 months.

The results are shown in Table III. No tumours developed in any of the S low-breast-cancer strain females, whether injected with RIII or 63 Ca dried breast tumour tissue. These females lived to an average age of 17·8 months.

In the F1 generation of S strain females injected with 63 Ca tissue and of the control females, no tumours were observed in any of the females, or in any of the males painted with oestrone. In the F1 generation of S strain females injected with RIII dried tumour tissue, 7 out of 16 females developed breast cancer, and 5 out of 15 males, which were painted, also developed breast tumours.

In S × RIII hybrid females obtained from daughters of S strain females injected with dried RIII breast tumour tissue, 27 out of 32 females showed breast tumours, while S × RIII hybrid males of the same origin, painted with oestrone, gave tumours in 10 out of 16 mice. A breast tumour developed in 1 out of 32 S × RIII hybrid females derived from daughters of S strain females injected with dried 63 Ca breast tumour tissue, and no tumours in any of the 22 S × RIII males painted. In S × RIII hybrid females of the third group 3 out of 34 developed breast cancer, and in 2 out of 32 S × RIII hybrid males of the same group, which were painted, breast cancer was observed.

In this experiment the milk factor was transmitted to the progeny of adult S low-breast-cancer strain females which themselves did not develop breast cancer. This is of interest in view of the recent findings of Andervont (1945*b*), who observed that adult female mice of another low-breast-cancer strain (strain I) injected with the milk factor did not develop breast cancer, and did not transmit it to the progeny obtained by mating these females to high-breast-cancer strain males, while adult susceptible mice, after a single injection of the milk factor, did not develop breast cancer, but transmitted it to their progeny. Bittner (1942*b*, 1943, 1944) noted a difference in the tumour incidence of successive litters born to the same susceptible females, and mice belonging to the third and fourth litters showed a higher tumour incidence than those of the first litters. It would have been of interest to compare the incidence of breast cancer in the first and the third litters of S strain females injected with RIII tumour tissue to see if any difference in the tumour incidence could be noted. This could not be done owing to the limited space available and the large numbers of mice it would involve. Bittner (1942*a*) observed that low-breast-cancer strain females fostered by high-breast-cancer strain mothers could transmit the milk factor in their milk, although they themselves did not develop breast cancer. In the present experiment, it was noted that the occurrence of breast cancer in S × RIII mice of the first group was independent of the appearance of a tumour in their F1 S strain mothers. The increased tumour incidence in S × RIII hybrid mice of the first group is another instance of the interaction of the milk factor, genetic constitution, and the hormonal factor in the development of breast cancer in mice. The addition of susceptible strain chromatin from RIII strain males resulted in mice developing more tumours.

Thus the present experiment has shown that the milk factor can be transmitted through relatively resistant strain S mice and induce a high incidence of breast cancer in susceptible mice. Again, it would have been of interest to observe the incidence of breast cancer in S × RIII hybrid mice obtained from S strain females injected with RIII dried tumour tissue, and to compare it with that of

S × RIII hybrid mice obtained from mating F1 S strain females. This could not be done owing to the numbers of mice it would involve.

It seems that mice of some, at least, low-breast-cancer strains like mice of high-breast-cancer strains may differ in their susceptibility to the action of the milk factor and also in their ability to transmit and propagate it. The ability to transmit the factor may vary from 100 per cent like in the C3H low-cancer strain of Andervont (1945*b*), through C57 strain of Fekete and Little (1942), CBA strain of Miller and Pybus (1945), down to strain I of Andervont (1945*a*).

Thus the presence of two sets of genetic factors, one controlling the susceptibility of breast tissue cells to the milk factor either directly or through the hormonal mechanism, the other controlling the propagation of the milk factor, must also be considered in low-breast-cancer strain mice.

The low incidence of breast cancer observed in S × RIII hybrid females forcibly bred, and in S × RIII males painted with oestrone, whether derived from the progeny of S low-cancer strain control females or S strain females injected with dried 63 Ca tumour tissue, has been previously described (Dmochowski, 1944*b*). It would have been of interest to test these tumours for the presence of the milk factor. Similar observations on hybrid mice derived from low-breast-cancer strain females and high-breast-cancer strain males have been reported from several sources (Murray and Little, 1939; Bagg and Jacksen, 1937; Bittner, 1939; Strong, 1943; Andervont, 1945*c*; Foulds, 1947). There was no significant difference in the incidence of breast cancer in hybrid mice from the S strain control mice, and in hybrid mice from the S strain females injected with dried 63 Ca tissue. It seems, therefore, that an increased hormonal stimulation rather than any possible small amount of the milk factor in 63 Ca tumour tissue may play a part in the development of these tumours.

*Experiment No.* 4.

The absence of the milk factor in 63 Ca transplantable tumour is shown in Table IV. No tumours developed in C57 × RIII hybrid females injected with

TABLE IV.—*Induction of Breast Cancer in C*57 × *RIII Hybrid Females by Administration of Dried RIII Breast Tumour and Dried* 63 *Transplantable Breast Tumour Tissue.*

| Number of mice. | Age of mice in weeks when first injected. | Number of injections. | Origin of breast tumour tissue. | Incidence of tumours (%). | Average tumour age in months. | Average age of mice without tumours in months. |
|---|---|---|---|---|---|---|
| 16 | 4–6 | 16 | 63 Ca | 0 | .. | 16·9 |
| 36 | 4–6 | 1 | RIII | 82·1 | 9·6 | 17·8 |

large doses of 63 Ca dried tissue, corresponding to 1·6 g. of fresh tissue, while their litter-mate controls injected with dried RIII breast tumour tissue (amount corresponding to 0·125 g. of fresh tissue) developed a high incidence of breast cancer. The presence of even small amounts of milk factor should have been observed by the method employed in the present experiment. It is impossible to state, however, whether the milk factor was originally absent in the primary tumour or whether it was lost during the course of so many transplantations.

In conclusion, it can be stated that only an analysis of the action and interaction of all known factors with allowance for other possible factors can make the solution of the origin of breast cancer in mice possible. All these factors play their part in the genesis of breast cancer in mice and it would be, at least, imprudent to state which is the most important.

### SUMMARY.

Differences in quantity and/or quality of the milk factor derived from two different high-breast-cancer strains have been shown and referred to differences in the genetic constitution of these strains.

Large quantities of the milk factor can break down the resistance of mature susceptible mice and induce a high incidence of breast cancer in these mice, while similar amounts of the milk factor combined with forced breeding have failed to induce breast cancer in mature mice of a low-breast-cancer strain.

Adult mice of another low-breast-cancer strain, when given the milk factor, have failed to develop breast cancer, but have transmitted the factor and have induced a high incidence of breast tumours in susceptible mice.

The interaction of milk factor and genetic constitution and the presence of two sets of genetic factors, one controlling the susceptibility of mice to the milk factor, the other the propagation of the factor, both in high- and low-breast-cancer strains, is discussed.

The possibility of the development of breast tumours in susceptible mice without the milk factor has been shown.

### REFERENCES.

ANDERVONT, H. B.—(1943) *J. nat. Cancer Inst.*, **3**, 359.—(1945*a*) *Ibid.*, **5**, 397.—(1945*b*) *Ibid.*, **5**, 383.—(1945*c*) *Ibid.*, **5**, 391.

*Idem* AND MCELENEY, W. J.—(1941) *Ibid.*, **1**, 737.

*Idem*, SHIMKIN, M. B., AND BRYAN, W. R.—(1942) *Ibid.*, **3**, 309.

BAGG, H. J.—(1939) 'Int. Cancer Res. Foundation, Report of Activities during 1939,' p. 14.

*Idem* AND JACKSEN, J.—(1937) *Amer. J. Cancer*, **30**, 539.

BITTNER, J. J.—(1936) *Proc. Soc. exp. Biol. N.Y.*, **34**, 42.—(1939) *Publ. Hlth. Rep., Wash.*, **54**, 1113.—(1940) *J. nat. Cancer Inst.*, **1**, 155.—(1942*a*) *Cancer Res.*, **2**, 710.—(1942*b*) *Ibid.*, **2**, 540.—(1943) *Ibid.*, **3**, 441.—(1944) *Ibid.*, **4**, 159.—(1945) 'A.A.A.S. Res., Conference on Cancer,' publ. A.A.A.S., Washington 25, D.C., p. 63.

*Idem* AND HUSEBY, R. A.—(1946) *Cancer Res.*, **6**, 235.

DMOCHOWSKI, L.—(1944*a*) *Brit. J. exp. Path.*, **25**, 138.—(1944*b*) *Ibid.*, **25**, 119.—(1945*a*) *Ibid.*, **26**, 192.—(1945*b*) *Ibid.*, **26**, 267.

*Idem* AND GYE, W. E.—(1943) *Ibid.*, **24**, 223.

FEKETE, E., AND LITTLE, C. C.—(1942) *Cancer Res.*, **2**, 525.

FOULDS, L.—(1947) *Brit. J. Cancer*, **1**, 362.

HESTON, W. E., AND ANDERVONT, H. B.—(1944) *J. nat. Cancer Inst.*, **4**, 403.

*Idem*, DERINGER, M. K., AND ANDERVONT, H. B.—(1945) *Ibid.*, **5**, 289.

KHANOLKAR, V. R., AND RANADIVE, K. J.—(1947) *J. Path. Bact.*, **59**, 593.

LITTLE, C. C., AND PEARSONS, J.—(1940) *Amer. J. Cancer*, **38**, 224.

MILLER, E. W., AND PYBUS, F. C.—(1945) *Cancer Res.*, **5**, 94.

MORRIS, H. P.—(1945) 'A Symposium on Mammary Tumours in Mice,' publ. A.A.A.S., by Members of the Staff of the Nat. Cancer Inst., Washington 25, D.C., p. 140.

MURRAY, W. S.—(1941) *Cancer Res.*, **1**, 123.

*Idem* AND LITTLE, C. C.—(1939) *Amer. J. Cancer*, **37**, 536.

STRONG, L. C.—(1943) *Proc. Soc. exp. Biol. N.Y.*, **53**, 257.

WARNER, G. G., REINHARD, M. G., AND GOLTZ, H. L.—(1945) *Cancer Res.*, **5**, 584.